# 

**Published:** 1904-06-04

**Authors:** 


### NOTICE TO CORRESPONDENTS.

All MSS., Letters, Books for Review, and other matters intended for the Editor should be addressed THE EDITOR, "THE HOSPITAL," THE HOSPITAL BUILDINGS, 28 & 29 SOUTHAMPTON STREET, STRAND, W.C.

The Editor cannot undertake to return rejected MS., even when accompanied by stamped directed envelope.

**Notice to Advertisers and Subscribers.**

All Advertisements, Orders for Copies of THE HOSPITAL, and Business Communications should be addressed to **THE MANAGER (Not to the Editor),** THE HOSPITAL, 28 & 29 Southampton Street, Strand, London, W.C.

The Cost of Subscription, post free, is as follows:—Per week, 3½d.; per quarter, 3s. 3d.; per half-year, 6s. 6d.; per annum, 13s. Foreign and Colonial:—Per annum, 19s. 6d., payable, in all cases, in advance.

**NOTICE.—Vol. XXXV. of "The Hospital" (October 1903 to March 1904), in handsome cloth binding, is now on sale at the office of this Journal, price 10s. 6d. Cases for binding the Numbers contained in this Volume 2s. Index to Vol. XXXV., 3½d., post free.**

**The Monthly part for June is now ready. Price 1s., by post, 1s. 3d.**

### MEDICAL APPOINTMENTS.

**George W. Badgerow**, M.B., M.R.C.S.Eng., L.R.C.P.Lond., Resident Medical Officer at the Throat Hospital, Golden Square. **Vivian Chastel de Boinville**, M.B., Ch.B.Edin., Assistant Director of Cancer Research, Liverpool University. **J. Jackson Clarke**, M.B.Lond., F.R.C.S., Surgeon to the North-west London Hospital. **W. E. Cooper**, M.R.C.S., L.R.C.P., District Medical Officer of the Thetford Union. **P. H. Court**, L.S.A., District Medical Officer of the Mansfield Union. **J. P. I. Harty**, M.B., B.Ch.R.U.I., District Medical Officer of the Halifax Union. **A. J. Hayes**, L.S.A.,Lond., International Quarantine Board of Egypt, posted to Suez and Moses Wells, to date from April 5th, 1904. **H. F. Hine**, M.B.Lond., F.R.C.S.Eng., Infirmary and District Medical Officer of the Newark Union. **John Alexander Mackenzie**, M.B., B.Ch.Aberd., Resident Medical Officer at the Oldham General Infirmary. **D. Moodie**, L.R.C.P.&S.Edin., Certifying Factory Surgeon for Coxhoe District, County Durham. **U. I. O'Kane**, M.B., B.Ch., B.A.O.R.U.I. Medical Officer for the Cushendall and Waterfoot Dispensary Districts, Surgeon to Cushendall Coastguard Station, and Medical Attendant to the Cushendall and Knocknacarry Police Stations. **C. F. Seville**, M.B.Lond., M.R.C.S., Certifying Factory Surgeon for the Rothwell District, County York. **W. Knowsley Sibley**, M.D.Camb., M.R.C.P., Physician to the North-West London Hospital. **G. A. Sutherland**, M.D.Edin., F.R.C.P., Physician to the North-West London Hospital. **George Templeton**, M.B.Edin., F.R.C.S., Surgeon to the North-West London Hospital. **A. M. Ware**, M.D.Cantab., Clinical Assistant to the Chelsea Hospital for Women. **Francis Kenneth Wilson**, M.B., B.S.Lond., M.R.C.S.Eng., L.R.C.P.Lond., District Medical Officer and Public Vaccinator, Walsham-le-Willows District of the Stow Union.

**"The Hospital" Institutional Library.**

"Hospitals and Asylums of the World." 4 Vols., with a Portfolio of Plans, complete £12 12s.

"Burdett's Hospitals and Charities, 1904." 5s. net.

"Burdett's Official Nursing Directory." 8s. net.

"Cottage Hospitals, General, Fever, and Convalescent." 10s. 6d.

"Uniform System of Accounts for Hospitals and Public Institutions." New Edition. 4s. net.

"Hospital Expenditure: The Commissariat." 2s. 6d.

"A Legal Handbook for the Use of Hospital Authorities." 2s. 6d.

"The Cottage Hospital Case Book or Register of Patients." 10s. and 12s. 6d.

All these are published by the SCIENTIFIC PRESS, Ltd., and may be obtained through any bookseller, or direct from the publishers, 28 and 29 Southampton Street, Strand, London, W.C.

### IMPORTANT HANDBOOKS ON MIDWIFERY.

*READY SHORTLY.*

### How to Become a Midwife

**And the Legal Requirements under the Midwives Act.**

**By E. L. C. APPEL, M.B., B.S., B.Sc.Lond.**

In this work the legal requirements of the Midwives Act are all set forth in such a way that any woman can by referring to the Table of Contents readily see which of them are applicable to her case. A List of the Training Schools recognised by the Central Midwives Board are also included in the book.

Crown 8vo. bound in limp cloth, 1/-; 1/1 post free.

### A Complete Handbook of Midwifery

**By J. K. WATSON, M.D., M.B., C.M.**

This handbook has been designed to meet the requirements of the Central Midwives Board, and is an up-to-date text-book of **all** that the midwife requires to know of the subject treated. The work is produced with the object of rendering it unnecessary for midwives, during their preparation for examination, to consult the larger and more expensive works on the subject written especially for medical men.

**The illustrations are very complete, numbering some 143 separate diagrams, most of which have been specially prepared for this book.**

Crown 8vo. about 350 pp., bound in cloth boards, 6/- net, 6/4 post free.

### The Midwives' Pocket Book

**By HONNOR MORTEN.**

**With Appendix containing particulars and Rules of the Central Midwives Board.**

Small crown 8vo. in cloth boards, 1/- post free.

### Nursing Notes on Midwifery and Gynaecology

**By SISTER ROSS,** Rotunda Hospital, Dublin.

Demy 16mo. cloth (suitable size for apron pocket), 2/-; 2/1 post free.

**Complete Catalogue of Nursing Manuals post free on application.**

*Of all Booksellers or of*

**THE SCIENTIFIC PRESS, LTD.,** 28 & 29 Southampton Street, Strand, LONDON, W.C.

